# Alveolar Soft Part Sarcoma of the Extremity: Case Report and Literature Review

**DOI:** 10.14740/wjon777w

**Published:** 2014-03-11

**Authors:** Khaled J. Ata, Hani N. Farsakh, Anwar Rjoop, Ismail Matalka, Liqa A. Rousan

**Affiliations:** a Department of Orthopedics, Jordan University of Science and Technology, King Abdullah University Hospital, Irbid, Jordan; b Department of Pathology, Jordan University of Science and Technology, King Abdullah University Hospital, Irbid, Jordan; c Department of Radiology and Nuclear Medicine, Jordan University of Science and Technology, King Abdullah University Hospital, Irbid, Jordan

**Keywords:** Alveolar soft part sarcoma, Extremity sarcoma

## Abstract

Alveolar soft part sarcoma (ASPS), a rare soft tissue sarcoma in children and adolescents, carries a poor prognosis. ASPS is an aggressive tumor of controversial histogenesis that, unlike other soft tissue sarcomas, tends to metastasize to the brain. A 9-year-old boy presented to our outpatient clinic in April 2009 with a chief complaint of a large painless mass in the left thigh whose size had increased significantly over the past 10 months. After staging the tumor, we performed open biopsy; the diagnosis was ASPS and he underwent wide local excision. In the course of 4-year follow-up by clinical and imaging studies, there was no evidence of early tumor recurrence or metastasis. Complete surgical resection is the treatment of choice in patients with ASPS.

## Introduction

Soft tissue sarcoma is a type of malignant soft tissue tumor that is usually linked to the tissue of origin. In alveolar soft part sarcomas (ASPS), the alveolar description is based on the histological pattern of the tumor. ASPS, a rare, malignant soft tissue neoplasm that affects young patients, accounts for approximately 0.5-1.0% of all soft tissue sarcomas [1] and for 5.0% of pediatric non-rhabdomyosarcoma soft tissue sarcomas [2]. While it usually presents as a painless mass in the extremities, these tumors have been reported to arise in other parts of the body [3]. Compared to other sarcomas, they are characterized by an unusual pattern of metastatic spread; brain metastasis is a common feature [4].

## Case Report

**Figure 1:**
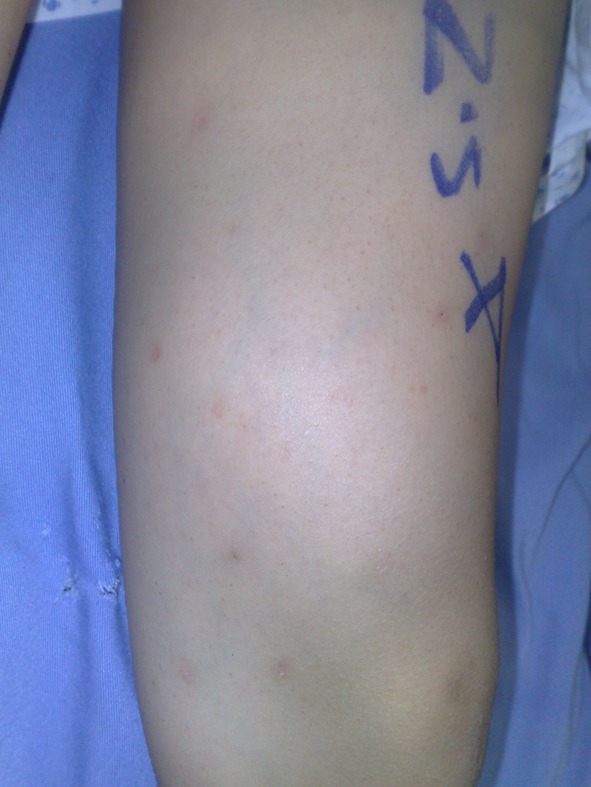
Palpable mass at inner aspect of distal thigh.

This 9-year-old boy presented to our outpatient clinic in April 2009 with a chief complaint of a painless large mass in the left thigh. He and his parents first noticed it as a small lump that increased in size over the next year till he visited our hospital. On examination, a mass measuring about 10 cm in diameter was noted at the anteromedial aspect of his left thigh about 15 cm above the knee joint. On palpation, it was firm and immobile. The mass was neither tender nor hot and there were no changes in the overlying skin (Fig. 1).

**Figure 2:**
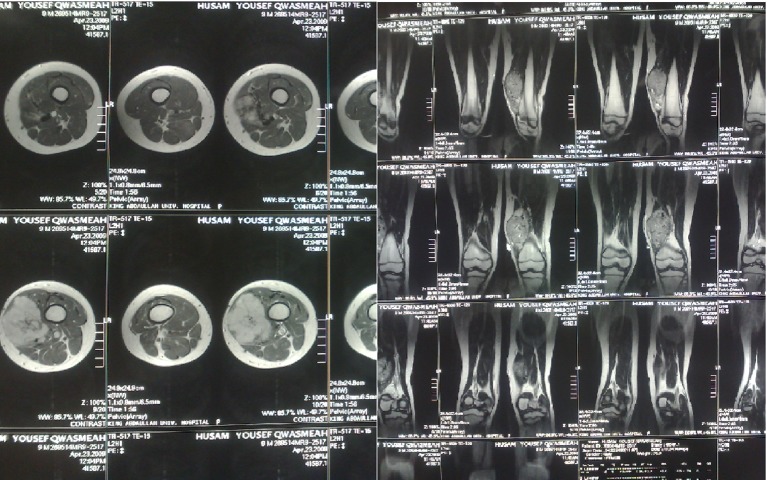
Pre-operative MRI findings.

Magnetic resonance imaging (MRI) of the left thigh, performed later in the same week, revealed a well-defined soft tissue mass, heterogeneously enhanced, measured 6.5 × 4 × 10 cm, displaced and invaded the lower vastus medialis muscle. There was no involvement or invasion of underlying bones and no evidence of a periosteal reaction (Fig. 2). The superficial femoral vein and artery were displaced laterally.

**Figure 3:**
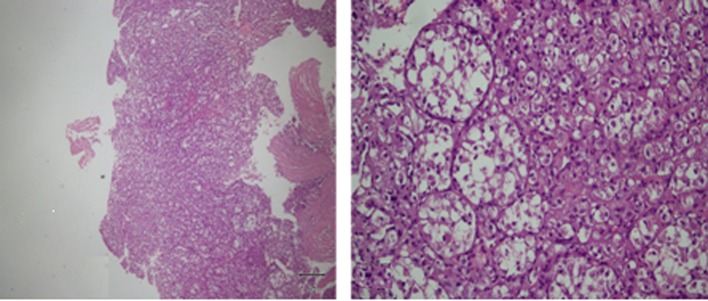
H&E of the incisional biopsy shows nested pattern arrangement of malignant cells which are large, polygonal at higher magnification.

These findings were suggestive of a heterogeneous soft tissue mass, possibly a sarcoma. Although a close biopsy is a realistic option, we did an open incisional biopsy, performed through a medial longitudinal incision centralized over the mass; it revealed multiple fragments of white soft tissue. Microscopic study showed sheets of malignant cells arranged in a nested pattern. They were large, polygonal with distinct cell borders and eosinophilic cytoplasm; they were separated by thin-walled vascular channels (Fig. 3).

**Figure 4:**
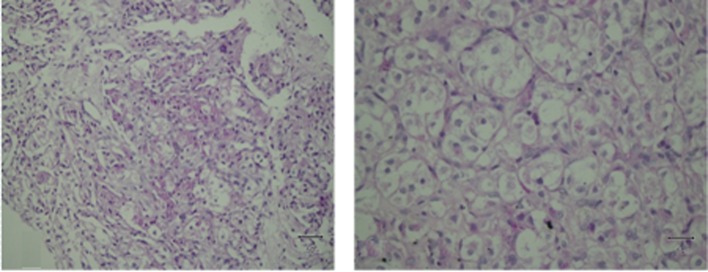
PAS/diastase stain positive for intracellular rod-shaped crystalloids.

The tumor cells contained intracellular crystals that were PAS-positive and diastase-resistant (Fig. 4). Immunostaining was negative for pan-cytokeratin, actin, desmin, myogenin, chromogranin, HMB45, melan A, vimentin, inhibin and S100. Based on these findings, a diagnosis of ASPS was made.

On whole-body bone (Tc99m), enhanced chest, abdominal, pelvic computed tomography (CT) and brain MRI scans, there was no evidence of distant metastasis.

**Figure 5:**
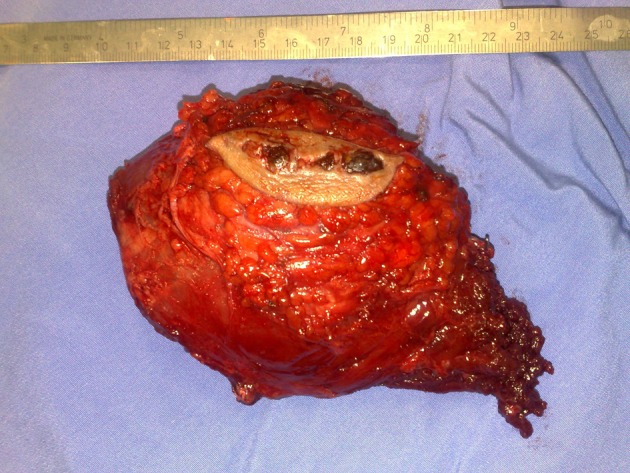
Soft tissue tumor after excision with biopsy tract.

After complete radiological and histological studies, he underwent wide local excision via an elliptical surgical incision to include the scar and tract of the earlier biopsy. Then we identified major vessels and performed a wide resection taking care not to expose the tumor. On gross examination, the tumor was a 12 × 8 × 6 cm mass with an ellipse of skin on its surface (Fig. 5).

The results of microscopic examination were similar to those of biopsy. All surgical margins were free; the closest was located 4 mm medially.

The postoperative course was unremarkable. The patient was seen every 5 - 7 months for clinical and radiological follow-up by local and brain MRI, CT chest, abdominal and pelvic scans. In the course of 48 months, there have been no signs of recurrence or metastasis.

## Discussion

ASPS, first described in 1952 by Christopherson et al [5], is characterized by an indolent course of slow growth and a high mortality risk. The tumor mainly affects the extremities with the thigh being the most common site although these tumors have been reported to arise in the trunk, the head and neck, and the tongue.

Metastasis usually occurs late in the course of the disease and involves the lungs, bones, lymph nodes and brain. Metastasis to the liver has also been reported [6]. To rule out brain metastasis, the Surgical Society of Oncology recommends intracranial imaging in all patients with ASPS [7].

There is some controversy over the pathogenesis of ASPS. While Mukai et al [8] provided support for a myogenic origin, Marchac et al [9] reported ASPL-TFE3 transcript fusion produced by chromosomal translocation (X; 17). According to Ordonez [10], there is a large female preponderance among ASPS patients. On the other hand, in their retrospective study, Portera et al [11] detected no significant gender difference when the patient population was considered as a whole although when they assessed patients with localized disease separately they also found a definite (64%) female preponderance.

The histological characteristics of ASPS include organoid nests of polygonal tumor cells encompassed by a dense capillary vasculature. The cells are of uniform size and shape and contain granular, eosinophilic cytoplasm surrounding a vesicular nucleus that hosts a prominent nucleolus. The designation “alveolar” derives from the pseudo-alveolar appearance [12].

While the role of radio- and chemotherapy is yet to be established, surgical resection with margins microscopically free of tumor cells remains the ASPS treatment of choice and represents a strong indicator of the treatment outcome [13]. Spontaneous regression has been reported [14].

According to Lieberman et al [12] who followed 102 patients over 63 years, the survival rate of patients with no metastasis at the time of diagnosis decreased dramatically from 77% at 2 years to 60% at 5 years, 38% at 10 years and 15% at 20 years. This indicates that periodic follow-up is mandatory in all patients with ASPS.

### Conclusion

In conclusion, ASPS is a rare type of sarcomas that affects primarily the lower limbs. Periodic radiologic follow-up is mandatory to detect metastasis. While the role of radio- and chemotherapy is yet to be established, complete surgical resection is the treatment of choice in patients with ASPS.
